# Effects of Continuous Positive Airway Pressure Therapy on Nocturnal Blood Pressure Fluctuation Patterns in Patients with Obstructive Sleep Apnea

**DOI:** 10.3390/ijerph19169906

**Published:** 2022-08-11

**Authors:** Hajime Kumagai, Hiroyuki Sawatari, Tetsuro Hoshino, Noriyuki Konishi, Yuka Kiyohara, Kengo Kawaguchi, Yoko Murase, Ayako Urabe, Aki Arita, Toshiaki Shiomi

**Affiliations:** 1 Department of Sleep Medicine, Graduate School of Biomedical and Health Sciences, Hiroshima University, Hiroshima 7348553, Japan; 2 Hiroshima Minato Clinic, Hiroshima 7340014, Japan; 3 Department of Perioperative and Critical Care Management, Graduate School of Biomedical and Health Sciences, Hiroshima University, Hiroshima 7348553, Japan; 4 Department of Sleep Medicine and Sleep Disorders Center, Aichi Medical University Hospital, Nagakute 4801195, Japan; 5 Department of Psychology and Medical Science, Graduate School of Psychology and Medical Sciences, Aichi Shukutoku University, Nagakute 4801197, Japan

**Keywords:** blood pressure fluctuation pattern, continuous positive airway pressure, nocturnal blood pressure fluctuations, obstructive sleep apnea, pulse transit time

## Abstract

This retrospective study was designed to evaluate the effects of continuous positive airway pressure (CPAP) therapy, a well-established treatment for obstructive sleep apnea (OSA), on nocturnal blood pressure fluctuations (NBPFs) during rapid eye movement (REM) and non-REM sleep, and to evaluate the NBPF patterns in patients with OSA. We included 34 patients with moderate-to-severe OSA who underwent polysomnography using pulse transit time before and at 3–6 months after CPAP therapy. Nocturnal BP and NBPF frequency in REM and non-REM sleep were investigated, as well as NBPF pattern changes after receiving CPAP therapy. CPAP therapy resulted in significant reductions in the apnea–hypopnea index (AHI), arousal index, nocturnal systolic and diastolic BP, and NBPF frequency in REM and non-REM sleep (all *p* < 0.01). A higher AHI before CPAP resulted in lower nocturnal systolic BP (*r* = 0.40, *p* = 0.019) and NBPFs (*r* = 0.51, *p* = 0.002) after CPAP. However, 58.8% of patients showed no change in NBPF patterns with CPAP therapy. CPAP therapy significantly improved almost all sleep-related parameters, nocturnal BP, and NBPF frequency in REM and non-REM sleep periods, but NBPF patterns showed various changes post-CPAP therapy. These results suggest that factors other than OSA influence changes in NBPF patterns.

## 1. Introduction

Obstructive sleep apnea (OSA) is a common disease that is globally recognized in several patients. The American Academy of Sleep Medicine (AASM) guidelines recommend continuous positive airway pressure (CPAP) therapy for patients with OSA and excessive daytime sleepiness [1]. Severe OSA is associated with cardiovascular disease (CVD), including hypertension [2,3,4,5,6]. A dose–response relationship between OSA severity and hypertension was also reported [7]. Moreover, the duration of CPAP therapy during sleep is related to its effectiveness in terms of sleepiness, daily functioning, hypertension, and CVD [4,5,6,8,9,10].

In patients with severe OSA, nocturnal blood pressure (NBP) undergoes several NBP fluctuations (NBPFs) [11]. OSA severity is also associated with BP fluctuations and sympathetic nerve activity [12]. Nondipping NBPF patterns have been more frequently observed in patients with OSA [5,10,13] and are a risk factor for hypertension and CVD. NBPF patterns are also likely to affect the prognosis of CVD in such patients [4,5,6,10]. During the sleep stage, apnea during rapid eye movement (REM) sleep was reported to lead to an increase in blood pressure (BP) and sympathetic activity in rats in approximately 2 weeks [14]. Even in healthy humans, sympathetic activity significantly increased during REM sleep [15]. Additionally, a significant correlation between OSA severity and nondipping NBPF patterns during non-REM sleep was reported [16].

Therefore, differences may exist in BP variability in response to respiratory events, such as apnea during REM and non-REM sleep. To the best of our knowledge, changes in the frequency of NBPFs during REM and non-REM sleep and NBPF patterns before and after CPAP therapy in patients with OSA have not been investigated. Therefore, we aimed to investigate changes in NBP, frequency of NBPFs during REM and non-REM sleep using pulse transit time (PTT), and NBPF patterns after CPAP therapy in patients with moderate-to-severe OSA.

## 2. Materials and Methods

### 2.1. Study Patients

In this retrospective study using nonprobability sampling, simultaneous PTT measurements and polysomnography (PSG) were performed twice: at the time of OSA diagnosis and post-CPAP therapy. Changes in sleep-related parameters and NBPFs before and after CPAP therapy were investigated. This study comprised 34 patients (men, *n* = 25 (73.5%)) who underwent PSG at the Hiroshima Minato Clinic between November 2018 and June 2019 and who had been diagnosed with OSA (apnea–hypopnea index (AHI) ≥ 20/h), initiated CPAP therapy, and were available to retest for PSG at 3–6 months after starting CPAP therapy. In Japan, patients with AHI ≥20/h are covered by medical insurance for CPAP therapy. All included patients had undergone PSG and had been assessed for NBP, frequency of NBPFs, and NBPF patterns using the PTT before and after CPAP therapy. Based on the recorded NBPF patterns, patients were categorized as having a dipping or nondipping pattern. All data were collected from available medical records. There were four smokers. All 12 patients on antihypertensive medication (men, *n* = 6) had commenced antihypertensive therapy prior to the first PSG, and the antihypertensive medication was not changed until the second PSG. With respect to complications, diabetes mellitus and dyslipidemia were observed in two and six patients, respectively. No patients had any complications or treatment history of atrial fibrillation. The exclusion criteria were as follows: (i) age < 20 and >80 years; (ii) treatment history of using CPAP or an oral appliance; and (iii) sleep disorders other than OSA. This study complied with the tenets of the Declaration of Helsinki and was approved by the Institutional Review Board of Aichi Medical University Hospital (approval number: 2021-049; approval date: 6 July 2021). All patients provided informed consent. As this was a retrospective study, an opt-out statement was used for patient consent, and none of the patients opted out.

### 2.2. PSG

All patients had undergone overnight PSG using SOMNOtouch™ RESP (SOMNOmedics, Randersacker, Germany) as a respiratory sleep screening device. PSG included electroencephalography; electrooculography; electromyography of the submental and anterior tibial muscles; electrocardiography; and nasal flow, thoracic and abdominal movement with respiratory effort, oxygen saturation, snoring, finger plethysmography, and body position analysis. The recorded data were analyzed using DOMINO light software v.1.4.0. (SOMNOmedics, Randersacker, Germany). PSG scoring was manually performed according to the American Academy of Sleep Medicine scoring criteria (version 2.3) by a certified sleep technologist blinded to the patient information. Apnea was scored upon cessation or a drop of airflow signal to ≤90% from the baseline for at least 10 s. Hypopnea was defined as a ≥30% reduction in airflow for at least 10 s associated with arousal or ≥3% oxyhemoglobin desaturation. The AHI and arousal index were calculated as the average number of apnea and hypopnea events per hour and the number of arousals per hour of sleep, respectively. The cumulative time percentage with SpO_2_ < 90% (CT90) was the cumulative time spent with oxygen saturation < 90%.

### 2.3. CPAP Therapy

CPAP therapy was performed using a CPAP device (S9 and AirSense10, ResMed Ltd., Bella Vista, NSW, Australia; or DreamStation, Philips Respironics, Murrysville, PA, USA). At the initiation of CPAP therapy, the physician explained in detail the importance of patient adherence to CPAP usage. The CPAP setting was adjusted according to the patient’s condition, and the device was used in automode. Daily usage status was confirmed through remote monitoring of the CPAP device (NemLink^®^, Teijin Pharma, Tokyo, Japan; f’Rens^®^, Fukuda Denshi, Tokyo, Japan; and Care Orchestrator^®^, Philips Japan, Tokyo, Japan). Patients were followed up at our clinic at 1–2 weeks after the start of CPAP therapy to confirm their use of the CPAP device, evaluate the adequacy of CPAP, and obtain insights into the impressions and challenges facing patients using the CPAP device. Subsequently, subjective symptoms and remotely monitored usage status were confirmed face-to-face or via telephone interviews monthly. No patients dropped out between the period of CPAP therapy initiation and a second PSG with CPAP. Good adherence was defined as a CPAP usage rate of ≥70%/month and an average CPAP usage time of ≥4 h/day.

### 2.4. BP Measurement

BP and frequency of NBPFs were calculated with DOMINO software using PTT, which was simultaneously performed with PSG. PTT is a well-established method for measuring BP. Significantly high correlations between BP measurements obtained using oscillometric and PTT-based methods have also been reported, which have confirmed the efficacy of PTT [17,18,19,20,21]. PTT-BP (i.e., BP obtained using PTT) has several advantages over 24 h ambulatory BP monitoring, such as noninvasive cuff-less measurement that does not disturb sleep, availability of beat-to-beat BP calculation and beat-to-beat BPF analysis, and detailed monitoring during sleep through continuous recording of beat-to-beat NBPFs. Therefore, PTT-BP can noninvasively recognize notable NBPFs due to OSA during sleep. PTT was calculated as the time taken for the R-wave on the electrocardiogram and the corresponding pulse wave to reach the finger plethysmograph. Systolic BP (SBP) and diastolic BP (DBP) were automatically estimated using DOMINO software, based on one initial BP calibration, using the oscillometric method on the upper arm [19,20,22]. Diurnal BP was measured using PTT and was obtained on an average of 3–4 h prior to falling asleep and at 1–2 h after waking up. NBPFs associated with respiratory events were defined as respiratory-related NBPFs. NBPF patterns were defined as: (i) dipping (≥10%), (ii) nondipping (from ≥0% to <10%), (iii) reverse dipping (<0%), and (iv) extreme dipping (≥20% fall from daytime to nighttime). The frequency of NBPFs was defined as the number of times per hour, in which SBP increased by ≥12 mmHg within 30 s during sleep.

### 2.5. Statistical Analysis

The primary outcome in this study was changes in BP after CPAP therapy in REM and non-REM sleep. The required sample size was estimated to be 32 (α = 0.05, power = 80%) to 42 (α = 0.05, power = 90%) individuals [23]. All statistical analyses were performed using JMP software version 16.0.0 (SAS Institute Japan, Tokyo, Japan). Continuous variables are expressed as means ± standard deviation, whereas categorical variables are presented as numbers or percentages. To compare differences in pre- and post-CPAP conditions with respect to demographic, polysomnographic, and PTT parameters, paired *t*-tests and Wilcoxon rank-sum tests were conducted for continuous variables. For univariate regression analysis, we estimated Spearman’s rank or Pearson’s correlation coefficients. All comparisons were two-tailed, and a *p*-value < 0.05 was considered statistically significant.

## 3. Results

### 3.1. Demographics and Effects of CPAP Therapy on Sleep-Related Parameters

Demographic characteristics and sleep-related parameters are presented in Table 1. The mean age and body mass index of patients with OSA were 50.5 ± 10.4 years and 26.5 ± 5.2 kg/m^2^, respectively. The mean AHI, arousal index, and CT90 were 39.3 ± 18.1/h, 22.7 ± 14.1/h, and 16.1 ± 27.7/min, respectively. The CPAP usage rate/month and average CPAP usage time/day were 86.0% and 4.9 h, respectively, which indicated good adherence to CPAP therapy.

**Figure 1:**
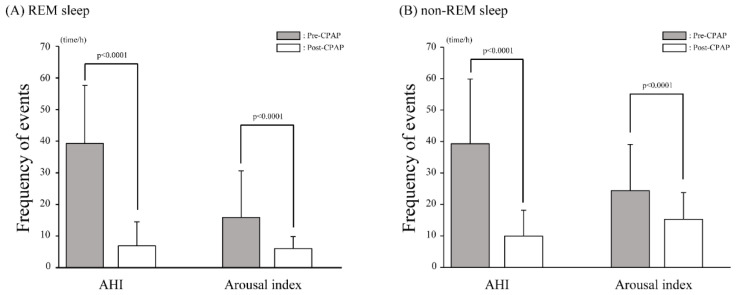
A comparison of sleep-related parameters pre- and post-CPAP. (**A**) REM sleep. (**B**) Non-REM sleep. Gray and white bars indicate the values pre- and post-CPAP, respectively. Values are presented as means ± SDs; error bars represent SD. AHI, apnea-hypopnea index; CPAP, continuous positive airway pressure; SD, standard deviation.

CPAP therapy significantly improved sleep-related parameters, except for the total sleep time, periodic limb movement index, sleep latency, and REM sleep latency. Furthermore, CPAP therapy significantly reduced the AHI and arousal index during REM and non-REM sleep (all *p* < 0.0001) (Figure 1A,B).

### 3.2. Effects of CPAP Therapy on NBP and NBPFs

**Figure 2:**
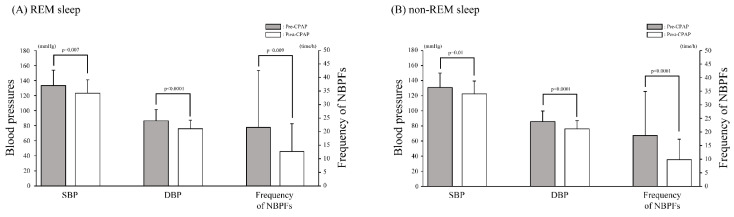
A comparison between blood pressures and NBPFs pre- and post-CPAP. (**A**) REM sleep. (**B**) Non-REM sleep. Gray and white bars indicate the values pre- and post-CPAP, respectively. Values are presented as means ± SDs; error bars represent SD. CPAP, continuous positive airway pressure; DBP, diastolic blood pressure; NBPFs, nocturnal blood pressure fluctuations; SBP, systolic blood pressure; SD, standard deviation.

SBP and DBP in REM and non-REM sleep were significantly reduced after CPAP therapy (SBP, *p* = 0.0045 and *p* = 0.0067; DBP, *p* = 0.0002 and *p* = 0.0003, for REM and non-REM, respectively) (Figure 2A,B). The average diurnal SBP and DBP were significantly reduced (*p* = 0.0079 and *p* = 0.0022, respectively). Similarly, the average nocturnal SBP and DBP were also significantly reduced (*p* = 0.0039 and *p* = 0.0004, respectively). The fluctuation ranges for BP before and after CPAP therapy were 6.1 and 9.0 mmHg for SBP during REM sleep (REM-SBP), 9.2 and 10.8 mmHg for DBP during REM sleep (REM-DBP), 3.8 and 9.6 mmHg for SBP during non-REM sleep (NREM-SBP), and 9.3 and 9.6 mmHg for DBP during non-REM sleep (NREM-DBP), respectively. However, the maximum increase in nocturnal SBP (Δ n-SBP) did not significantly change after CPAP therapy. The average diurnal heart rate was not reduced after CPAP therapy (*p* = 0.375), although the nocturnal heart rate significantly decreased (*p* = 0.0012) (Table 2). Before CPAP therapy, no statistically significant differences were observed between patients on or not on antihypertensive medication in terms of REM-SBP (140.3 ± 25.0 vs. 129.6 ± 16.7 mmHg, *p* = 0.1436, respectively), REM-DBP (92.2 ± 19.6 vs. 83.4 ± 11.0 mmHg, *p* = 0.1543, respectively), NREM-SBP (138.6 ± 22.6 vs. 126.8 ± 16.0 mmHg, *p* = 0.0851, respectively), and NREM-DBP (92.3 ± 17.9 vs. 82.0 ± 10.3 mmHg, *p* = 0.1298, respectively).

**Figure 3:**
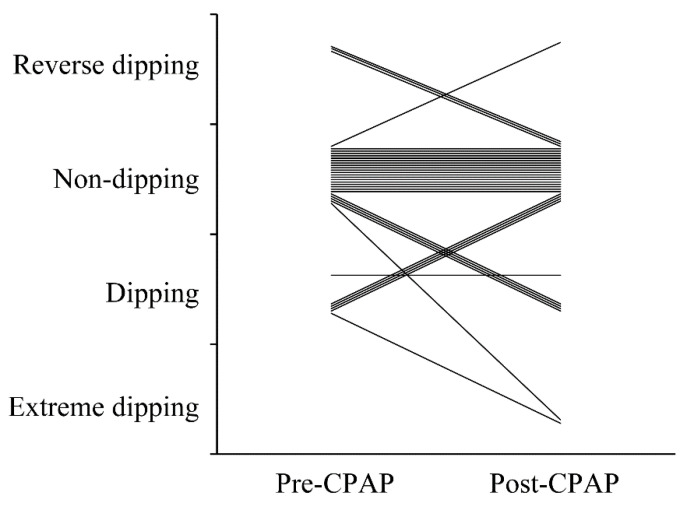
Changes in nocturnal blood pressure fluctuation patterns pre- and post-CPAP therapy. CPAP, continuous positive airway pressure.

The frequency of NBPFs was significantly reduced in REM and non-REM sleep after CPAP therapy (*p* = 0.009 and *p* < 0.0001, respectively) (Figure 2A,B). Respiratory-related NBPFs were also significantly reduced after CPAP therapy (*p* < 0.0001). Changes in NBPF patterns before and after CPAP therapy are shown in Figure 3. The prevalence of NBPF patterns such as dipping, nondipping, reverse-dipping, and extreme dipping pre- and post-CPAP ranged from 17.6% (*n* = 6) to 14.7% (*n* = 5), 73.5% (*n* = 25) to 76.5% (*n* = 26), 8.8% (*n* = 3) to 2.9% (*n* = 1), and 0% (*n* = 0) to 5.9% (*n* = 2), respectively. The NBPF patterns in 20 (58.8%) patients did not change, and only 5 (20.0%) among 25 patients with a nondipping pattern changed to a dipping or extreme-dipping pattern post-CPAP therapy. 

### 3.3. Association between Sleep-Related Parameters and Nocturnal BP

**Figure 4:**
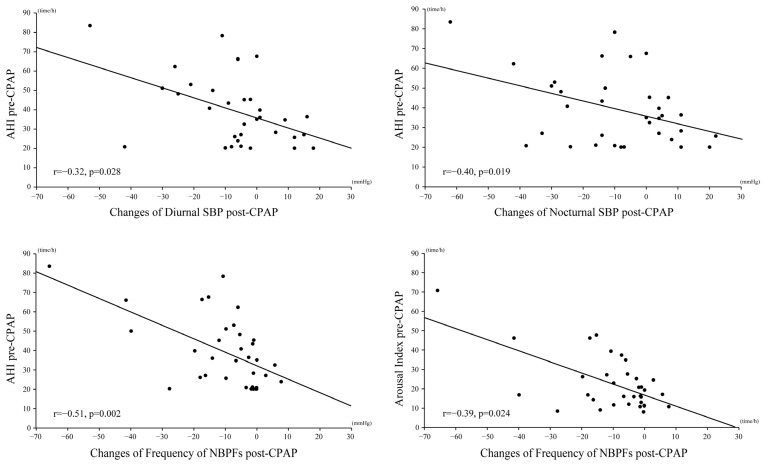
Correlation between sleep-related and nocturnal blood-pressure-related parameters. AHI, apnea-hypopnea index; CPAP, continuous positive airway pressure; DBP, diastolic blood pressure; NBPFs, nocturnal blood pressure fluctuations; SBP, systolic blood pressure.

A higher AHI pre-CPAP therapy was associated with a significant reduction in diurnal and nocturnal SBP and NBPFs post-CPAP (*r* = 0.32, *p* = 0.028; *r* = 0.40, *p* = 0.019; and *r* = 0.51, *p* = 0.002, respectively). In addition, a higher arousal index pre-CPAP was associated with a significant reduction in NBPFs post-CPAP (*r* = 0.39, *p* = 0.024) (Figure 4).

## 4. Discussion

The findings of this study revealed that CPAP therapy significantly reduced nocturnal SBP and DBP as well as the frequency of NBPFs during REM and non-REM sleep in patients with moderate-to-severe OSA. In addition, a higher AHI before CPAP therapy was associated with significantly reduced diurnal and nocturnal SBP and NBPFs after CPAP therapy. In contrast, changes in NBPF patterns after CPAP therapy varied and did not show a constant tendency.

The AASM guidelines note the importance of CPAP therapy for patients with OSA [1]. Long-term CPAP therapy has antihypertensive effects [8,23,24,25,26]. In this study, we revealed that CPAP therapy for 3–6 months significantly reduced SBP, DBP, and NBPFs during REM and non-REM sleep. Furthermore, we observed that diurnal SBP and DBP were also significantly reduced. CPAP therapy is considered effective if SBP and DBP are reduced by 2 and 1 mmHg, respectively [27]. In the present study, NBP was 9.2 mmHg lower after CPAP therapy than before CPAP therapy for both SBP and DBP, suggesting that CPAP therapy is effective as an antihypertensive therapy. Additionally, a decrease in NBPF during REM and non-REM sleep was detected. We observed that CPAP therapy was effective as an antihypertensive therapy, with additional reductions noted in NBPFs during REM and non-REM sleep.

Reducing nocturnal BP and NBPFs is crucial given the associated risk of cerebrocardiovascular and organ damage in patients with moderate-to-severe OSA [4,23,28,29]. Compared with non-REM sleep, REM sleep is associated with greater sympathetic activity and cardiovascular instability in healthy individuals and those with OSA [15,30,31]. In our study, CPAP therapy reduced nocturnal SBP and DBP as well as the frequency of NBPFs in both REM and non-REM sleep. Moreover, CPAP therapy reduced NBPFs, especially respiratory-related NBPFs. The reductions in nocturnal SBP and DBP suggests that CPAP may act via suppression of sympathetic nerve activation.

While CPAP therapy is effective against NBPFs from the first night of use [25], the results of the present study suggest that the positive effects on NBPFs may continue for 3–6 months after CPAP therapy. High SBP and DBP are known to increase the risk of CVD [32]. We suggest that the more severe the OSA, the greater the reductions in SBP, DBP, and frequency of NBPFs due to CPAP therapy, which may contribute to a reduced risk of CVD.

OSA is associated with NBPF pattern types, significantly increasing the risk of nondipping patterns [5,13]. Approximately 59% of patients with OSA have a nondipping pattern [10]. In addition, NBPFs and nondipping patterns are correlated with OSA severity [33]. A nondipping pattern is associated with hypertension, and CVD influences the overall prognosis due to CVD [4,5,6,10]. Patients with severe OSA have an increased risk of fatal and nonfatal CVD [34]. In our study, a nondipping pattern was observed in 82.4% and 79.4% of patients pre- and post-CPAP therapy, respectively. CPAP therapy significantly reduced the AHI as well as NBP and NBPFs; however, only 20.0% of the patients changed from a nondipping pattern to a dipping or extreme-dipping pattern with CPAP therapy. As OSA severity increases, the proportion of nondipping patterns and risk of CVD increase, suggesting the need for CPAP therapy to reduce such risk. Nonetheless, the effect of CPAP therapy on NBPF patterns was heterogeneous in this study. Thus, future studies should consider clarifying the causes of this heterogeneous effect and determine whether changes in NBPF patterns before and after CPAP therapy differ with respect to the risk of hypertension and CVD, as well as subsequent prognosis.

CPAP therapy is significantly associated with improved prognosis [35]. Therefore, CPAP should be used every night to maintain its therapeutic efficacy [8]. CPAP adherence is usually considered good when CPAP usage is ≥70%/month and the average usage time is ≥4 h/day [36]; however, if CPAP is used only for 3–4 h after the onset of sleep, 60–75% of REM-OSA persists [37]. In this study, CPAP therapy improved the AHI, arousal index, nocturnal SBP and DBP, and frequency of NBPFs in REM and non-REM sleep. CPAP therapy was also effective in controlling diurnal and nocturnal BP. Furthermore, the more severe the OSA, the greater the antihypertensive effect of CPAP therapy. Therefore, long-term and almost daily use of CPAP therapy during both REM and non-REM sleep is important for 24 h BP control and CVD prevention in individuals with greater OSA severity.

This study had some limitations. The patient sample size was small, and only Japanese patients were included. Therefore, it is possible that some items, such as maximum BP increase and changes in NBPF pattern, were not statistically significant. Further studies are needed to enroll a larger number of patients and examine changes in NBPF patterns after CPAP therapy in patients with a nondipping pattern diagnosed by PTT-BP at PSG diagnosis who are not taking antihypertensive medication, as well as the association between changes in NBPF patterns after long-term CPAP therapy and the development of CVD. As this was a retrospective study, we could not use randomization and blinding methods. We cannot deny any selected bias because of the retrospective nature of the study as we consecutively included patients with OSA. When considering PTT-BP, clinicians should be aware that sleeping position, snoring, and arrhythmia (e.g., atrial fibrillation) affect PTT-BP. The BP value used to calculate PTT-BP was only calibrated once; therefore, it may be preferable to evaluate BP fluctuations rather than absolute BP values. Furthermore, it should be noted that PTT is inversely proportional to BP values, and the decrease in BP was associated with inspiration prolonging the PTT [38]. Previous studies have shown that PTT-BP fluctuates as a result of inspiratory efforts and arousals [18,39]; nevertheless, the decrease in NBPFs after CPAP therapy suggests that CPAP may have suppressed inspiratory efforts and arousals. However, it was not clear whether inspiratory efforts or arousals had a more significant effect on the decrease in NBPFs. In diurnal SBP, CPAP therapy resulted in a significant reduction; however, PTT-BP measurements were limited to 3–4 h prior to falling asleep and 1–2 h after waking up. Thus, the fact that PTT-BP could not be assessed over a 24 h period should be taken into account when understanding the results of the present study. There is not a global consensus definition for evaluating NBPF yet. The definition of NBPFs, which we adopted in this study, might be useful to eliminate artifacts, such as pulsatory fluctuations, respiratory fluctuations, body position changes, and circadian rhythm. We hope that a consensus definition for the evaluation of NBPFs will be established as soon as possible. 

## 5. Conclusions

Nocturnal SBP and DBP as well as the frequency of NBPFs significantly improved after CPAP therapy during REM and non-REM sleep in patients with moderate-to-severe OSA. The varying NBPF patterns after CPAP therapy suggest that factors other than OSA may influence changes in NBPF patterns. This should be a point of caution in CVD prevention in patients with moderate-to-severe OSA.

## Figures and Tables

**Table 1:** Patient demographics and polysomnography.

	Pre-CPAP	Post-CPAP	*p*-Value
Sex (M/F (*n*))	M (*n* = 25) F (*n* = 9)	-	-
Age (years)	50.5 ± 10.4	-	-
Smoking (*n*)	4 (M: 4; F: 0)	-	-
Antihypertensive (*n*)	12 (M: 6; F: 6)	-	-
BMI (kg/m^2^)	26.5 ± 5.2	26.4 ± 4.3	0.761
*Sleep-related parameters*			
TST (min)	407.4 ± 81.2	422.0 ± 72.3	0.3042
SE (%)	82.6 ± 11.8	87.0 ± 7.6	0.0077 *
N1 (%)	29.0 ± 14.5	18.9 ± 8.4	<0.0001 *
N2 (%)	52.5 ± 13.2	56.7 ± 8.4	0.0135 *
N3 (%)	2.9 ± 4.6	4.0 ± 4.2	0.0152 *
REM (%)	15.6 ± 5.1	20.4 ± 5.7	<0.0001 *
AHI (/h)	39.3 ± 18.1	8.6 ± 6.1	<0.0001 *
REM-AHI (/h)	39.3 ± 18.3	6.9 ± 7.6	<0.0001 *
NREM-AHI (/h)	39.3 ± 20.5	9.9 ± 8.3	<0.0001 *
Arousal index (/h)	22.7 ± 14.1	13.3 ± 6.5	<0.0001 *
REM-arousal index (/h)	15.9 ± 14.7	6.0 ± 3.8	<0.0001 *
NREM-arousal index (/h)	24.4 ± 14.7	15.3 ± 8.4	<0.0001 *
Min SpO_2_ (%)	79.5 ± 8.6	86.2 ± 5.5	<0.0001 *
CT90 (min)	16.1 ± 27.7	0.9 ± 1.5	<0.0001 *
PLMI (/h)	4.1 ± 8.1	4.5 ± 9.6	0.4489
SL (min)	20.1 ± 29.5	14.1 ± 15.7	0.2066
REM-SL (min)	98.0 ± 73.0	92.7 ± 60.6	0.4455

Continuous variables are expressed as means ± standard deviations. Differences were compared pre- and post-CPAP. * *p* < 0.05. AHI, apnea-hypopnea index; BMI, body mass index; CPAP, continuous positive airway pressure; CT90, cumulative time percentage with SpO_2_ <90%; N1, sleep stage N1; NREM-AHI, AHI during NREM sleep; PLMI, periodic limb movement index; REM, rapid eye movement; SE, sleep efficiency; SL, sleep latency; TST, total sleep time.

**Table 2:** Changes in nocturnal BP fluctuations, BP, and heart rate.

	Pre-CPAP	Post-CPAP	*p*-Value
Frequency of NBPFs (/h)	20.8 ± 18.2	10.6 ± 7.5	<0.0001 *
Respiratory-related NBPFs (/h)	11.4 ± 13.8	2.3 ± 2.8	<0.0001 *
Maximum BP increase (mmHg)	30.8 ± 14.3	26.4 ± 9.8	0.0972
Diurnal average SBP (mmHg)	138.6 ± 18.5	131.9 ± 15.3	0.0079 *
Diurnal average DBP (mmHg)	91.8 ± 13.7	84.1 ± 10.1	0.0022 *
Diurnal average HR (bpm)	80.5 ± 9.4	80.0 ± 9.1	0.375
Nocturnal average SBP (mmHg)	131.8 ± 19.2	122.6 ± 17.7	0.0039 *
Nocturnal average DBP (mmHg)	86.3 ± 14.3	77.1 ± 11.2	0.0004 *
Nocturnal average HR (bpm)	69.7 ± 9.3	65.8 ± 9.1	0.0012 *

Continuous variables are expressed as means ± standard deviations. Differences were compared pre- and post-CPAP. * *p* < 0.05. CPAP, continuous positive airway pressure; DBP, diastolic blood pressure; HR, heart rate; bpm, beats per minute; NBPFs, nocturnal blood pressure fluctuations; SBP, systolic blood pressure.

## Data Availability

Data sharing is not applicable to this article.
